# Differential Effects of Aripiprazole and Amisulpride on Negative and Cognitive Symptoms in Patients With First-Episode Psychoses

**DOI:** 10.3389/fpsyt.2022.834333

**Published:** 2022-03-17

**Authors:** Mette Ødegaard Nielsen, Tina Dam Kristensen, Kirsten Borup Bojesen, Birte Y. Glenthøj, Cecilie K. Lemvigh, Bjørn H. Ebdrup

**Affiliations:** ^1^Center for Neuropsychiatric Schizophrenia Research (CNSR), Copenhagen University Hospital – Mental Health Services Copenhagen, Copenhagen, Denmark; ^2^Center for Clinical Intervention and Neuropsychiatric Schizophrenia Research (CINS), Copenhagen University Hospital – Mental Health Services Copenhagen, Copenhagen, Denmark; ^3^Mental Health Center, Glostrup, Copenhagen University Hospital – Mental Health Services Copenhagen, Copenhagen, Denmark; ^4^Department of Clinical Medicine, Faculty of Health and Medical Science, University of Copenhagen, Copenhagen, Denmark

**Keywords:** negative symptoms, cognitive deficits, antipsychotic treatment, dopamine antagonist, partial dopamine agonist

## Abstract

**Introduction:**

Aripiprazole is hypothesized to have an effect on negative and cognitive symptoms in schizophrenia. Likewise, amisulpride is one of the only second-generation antipsychotics with which an effect on negative symptoms is reported. In the present study, we compare the effect of aripiprazole and amisulpride in initially antipsychotic-naïve patients with first-episode psychoses.

**Methods:**

Psychopathology and cognitive measures from two consecutive cohorts of antipsychotic-naïve first episode psychotic patients were obtained before and after 6 weeks of antipsychotic monotherapy with either aripiprazole or amisulpride. Matched healthy controls were included to account for retest effects on the cognitive measures. Analyses of variance (repeated-measures ANOVA) were performed to detect effect of time and possible cohort^*^time interactions.

**Results:**

Longitudinal data was obtained from 47 and 48 patients treated for 6 weeks with amisulpride or aripiprazole, respectively. For the Wallwork negative symptom dimension, there was a cohort^*^time interaction [*F*_(1, 93)_ = 4.29, *p* = 0.041] and a significant effect of time [*F*_(1, 93)_ = 6.03, *p* = 0.016], which was driven by an improvement in patients treated with aripiprazole [*t*_(47)_ = 4.1, *p* < 0.001] and not observed in patients treated with amisulpride (*p* > 0.5). For the eight cognitive measures, no cohort^*^time interaction was found and neither was cognitive improvement in any of the cohorts when accounting for retest effect.

**Conclusion:**

Patients treated with aripiprazole improved on negative symptoms, which was not the case for patients treated with amisulpride. This may point to a general effect of a partial D2 receptor agonist on negative symptoms in patients with first-episode psychoses. There was, however, no improvement in cognitive functions.

## Introduction

Patients with schizophrenia often suffer from multiple symptoms. Antipsychotic medication ameliorates psychotic symptoms in most patients, but negative symptoms and cognitive deficits rarely improve during treatment (1–3). This constitutes a major clinical challenge because these symptoms are associated with worse outcomes in terms of lower levels of functioning and quality of life (4–6).

Psychotic symptoms are associated with a dopaminergic hyperactivity in ventral and associative parts of striatum (7–9). Although the neurobiological underpinnings of cognitive deficits and negative symptoms are not fully understood, they are both associated with disturbances in cerebral networks and may, to some degree, be related to a hypodopaminergic function in prefrontal cortex (10–12).

So-called first- and second-generation antipsychotic medication work by D2 antagonism thereby dampens an overactive dopamine turnover in the more ventral parts of striatum. Partial D2 agonists are denoted third-generation antipsychotics and are hypothesized to dampen the overactive dopamine system in striatal regions but increase dopamine-induced signaling in hypodopaminergic areas such as prefrontal cortex. Theoretically, this may improve negative symptoms and cognitive deficits (13).

Amisulpride is a relatively selective D2 receptor antagonist but is categorized as a second-generation antipsychotic because of a limbic selectivity (14). Due to an affinity for presynaptic D1 receptors in striatum, there should primarily be an effect on negative symptoms when given in doses below 300 mg, which has also been confirmed in two meta-analyses (15, 16). Few studies point to a small improvement in cognitive functions after treatment with amisulpride and other second-generation antipsychotics (17, 18); this may, however, primarily be caused by practice effects (19).

Aripiprazole was the first partial D2 receptor agonist registered for treating psychoses. Although it has been used for two decades, only a few studies focus on the effect on negative symptoms. In studies comparing the effect of aripiprazole to first-generation antipsychotics, aripiprazole showed a superior effect on negative symptoms (20, 21). Studies comparing aripiprazole with second-generation antipsychotics have primarily used risperidone and did not demonstrate a differential effect on the global negative symptom score (22–24) although a superior effect on the avolition-apathy subscore was found in one study (24).

Regarding a possible effect on cognitive impairments, a few open-label trials demonstrate a positive effect on verbal cognitive functions 8–26 weeks after switching to aripiprazole (25–27) although this was not the case in all studies (28). These studies were all carried out in patients who were already medicated and changed to aripiprazole from other antipsychotic drugs. One study examined the effect of using aripiprazole as adjunctive treatment and found a negative effect on verbal fluency and executive functions although motor speed was improved (29). None of the previous studies included a placebo or a healthy control group to correct for retest effects, which is of great importance in trials measuring cognitive functions (30). Further, there are no studies examining the effect of aripiprazole on cognitive functions in patients with first-episode psychoses.

In the Danish guidelines, both aripiprazole and amisulpride are recommended as first-line treatment for patients diagnosed with first-episode psychoses (31). Because both are also suggested to be effective for treating negative symptoms, we found it relevant to use the data from two consecutive cohorts of first-episode psychoses patients to compare their effect on negative symptoms. Based on the partial dopamine agonistic effect, we hypothesized that aripiprazole would show a superior effect on negative symptoms compared with amisulpride. Secondarily, we explored the effect on selected cognitive measures and hypothesized that patients treated with aripiprazole would improve in cognitive performance compared with patients treated with amisulpride.

## Materials and Methods

Data were collected in the Capital Region of Denmark, Copenhagen, as part of two consecutive longitudinal multimodal studies; the PECANS 1 cohort 2009–2013 (here denoted “amisulpride cohort”) and the PECANS 2 cohort 2013–2019 (here denoted “aripiprazole cohort”). Detailed descriptions of the studies can be found in (32, 33) and www.clinicaltrials.gov (NCT01154829, NCT02339844). For a full overview of previous publications, please see www.cinsr.dk. Participants provided oral and written informed consent prior to inclusion, and both studies were approved by the regional Committee on Biomedical Research Ethics (H-D-2008-088, H-3-2013-149).

### Participants

Patients were recruited from psychiatric hospitals and outpatient clinics in the Copenhagen catchment area. Diagnoses according to International Classification of Diseases 10th revision (ICD-10) were confirmed using the Schedules for Clinical Assessment in Neuropsychiatry (SCAN), version 2.1 (34). For the amisulpride cohort, patients met the criteria for schizophrenia (DF20.x) or schizoaffective psychoses (DF25.x), whereas patients with diagnoses in the non-affective psychotic spectrum (DF2X.x except schizotypal disorder, DF21.x) were also included in the aripiprazole cohort. All patients were strictly antipsychotic-naïve and had never been treated with methylphenidate, whereas treatment with antidepressant medication more than a month before the baseline examinations was accepted. Previous or present use of benzodiazepines was allowed. Other exclusion criteria were current diagnosis of drug dependency, involuntary admission or treatment, or severe physical illness. Current occasional use of substances and benzodiazepines and previous substance abuse was accepted for patients.

Two consecutive groups of healthy controls (HC) matched to patients based on age (±2 years), sex, and parental socioeconomic status were recruited using online advertisement. Exclusion criteria for HCs were any physical or mental illness, substance abuse, and having a first-degree relative with psychotic symptoms. Data from the HCs are in the present study only used for calculating *z*-values for the cognitive measures.

### Clinical and Cognitive Assessments

At baseline and after 6 weeks, psychopathology in patients was assessed using the Positive And Negative Syndrome Scale (PANSS) (35). Because we were particularly interested in the effect on negative symptoms and the original PANSS negative symptom cluster has been criticized (36–38), our primary outcome was the negative symptom dimension described by Wallwork et al. (39), which is also found to be most ideal among patients with first-episode psychosis (40). In the Wallwork five-factor model, the negative dimension includes the following items from the PANSS scale: N1: Blunted affect; N2: Emotional withdrawal; N3: Poor rapport; N4: Passive/apathetic social withdrawal; N6: Lack of spontaneity and flow of conversation, and G7; Motor retardation. Additional analyses were performed on the original PANSS negative, positive, and general end total PANSS-scores.

Level of functions was estimated with the Global Assessment of Function scale (GAF) (41), and adverse effects were estimated with the Extrapyramidal Symptom Rating Scale (ESRS) (42).

Cognitive functions were examined using the Cambridge Neuropsychological Test Automated Battery (CANTAB) (43, 44) and the Brief Assessment of Cognition in Schizophrenia (BACS) (45). We focused our analyses on verbal working memory (number sequences, NSq), verbal fluency (VF), and processing speed (symbol coding, SC) from BACS and measures of spatial working memory, (strategy and between errors from Spatial Working Memory [SWM]), planning (Stockings of Cambridge [SOC]), mental flexibility (Intra-Extradimensional Set Shifting [IED]), and sustained attention (A' from Rapid Visual Information processing [RVP]) from CANTAB.

For each cohort separately, the means and standard deviations of the HCs at both time points were used to calculate *z*-scores for patients, thereby accounting for retest effect. *Z*-scores for SWM and IED were inverted to report all variables in the same direction and ease the interpretation; i.e., a negative *z*-score indicates less successful performance in patients compared with HCs.

### Treatment

After baseline assessments, patients commenced treatment for 6 weeks with amisulpride or aripiprazole. The dose was individually adjusted according to the clinical impression of symptoms and report of adverse effects.

### Statistics

Information on demography and baseline psychopathology was compared using Chi square and independent *t*-tests. Repeated-measures ANOVA was used to evaluate cohort^*^time interaction for the primary outcome; the Wallwork negative dimension; and for the secondary outcome, the selected cognitive measures. To account for the multiple comparison effect of analyzing eight cognitive measures, the corrected significance threshold for secondary analyses was ≤ 0.006 (0.05/8). *Post hoc* analyses were performed using independent and paired *t*-tests.

Explorative analyses were performed on the original PANSS symptom clusters, GAF, ESRS, weight and BMI. Because of a small difference in sex distribution and age, primary analysis was performed with sex and age as cofactors.

To account for patients who did not complete the follow up, analyses were repeated using mixed modeling, and dropout analyses were done using one-way ANOVA.

Finally, we repeated analyses including only the patients with schizophrenia/schizoaffective psychoses from the aripiprazole cohort (*n* = 35).

## Results

**Figure 1:**
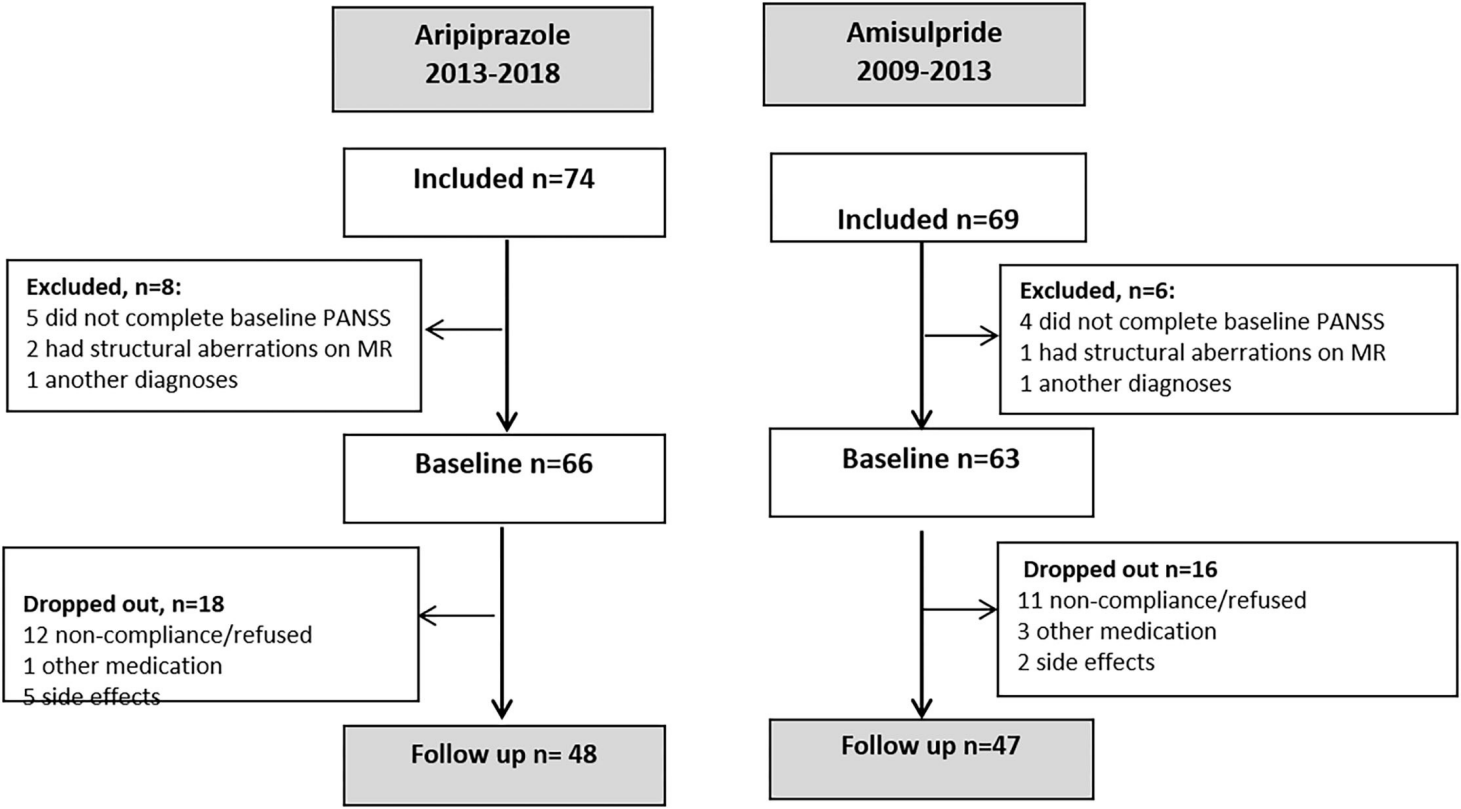
Flowcharts of the two studies.

In total, 69 and 74 patients were included in the two cohorts. Baseline and follow-up measures on psychopathology were obtained from 47 patients from the amisulpride cohort and 48 patients from the aripiprazole cohort; numbers and reasons for exclusion are illustrated in Figure 1. For patients who completed the study, there were no differences between cohorts in age, sex, or baseline level of psychopathology except from a higher PANSS general mean score in the amisulpride cohort. In the amisulpride cohort, 96% (*n* = 45) had a schizophrenia diagnosis, and the remaining 4% (*n* = 2) were diagnosed with schizoaffective psychoses. For the aripiprazole cohort, 71% (*n* = 34) had a schizophrenia diagnosis, 2% (*n* = 1) were diagnosed with a schizoaffective psychosis, and the remaining 27% (*n* = 13) were diagnosed with other non-affective psychoses (see Table 1). Mean dose of antipsychotic treatment at follow up was 276 (±173, range 50–800) mg for amisulpride and 10 (±4.7, range 2.5–25) mg for aripiprazole. Converted into chlorpromazine equivalent (46), the doses were comparable (216 vs. 201 mg).

**Table 1:** Demography, antipsychotic dose, and diagnoses for both cohorts.

	**Amisulpride** ***N* = 47**	**Aripiprazole** ***N* = 48**
Age (SD, range), years	24.5 (6; 18–43)	22.9 (4; 18–42)
Sex, female/male	20/27	24/24
Dose, mg (SD, range)	276 (173; 50–800)	10 (4.7; 2.5–25)
Chlorpromazine equivalent, mg	216 (124; 37.5–600)	201 (94; 50–500)
**Diagnoses**		
Schizophrenia	45	34
Persistent delusional disorder		2
Schizoaffective psychoses	2	1
Other nonorganic psychotic disorders		8
Unspecified nonorganic psychotic disorders		3

### Psychopathology

**Figure 2:**
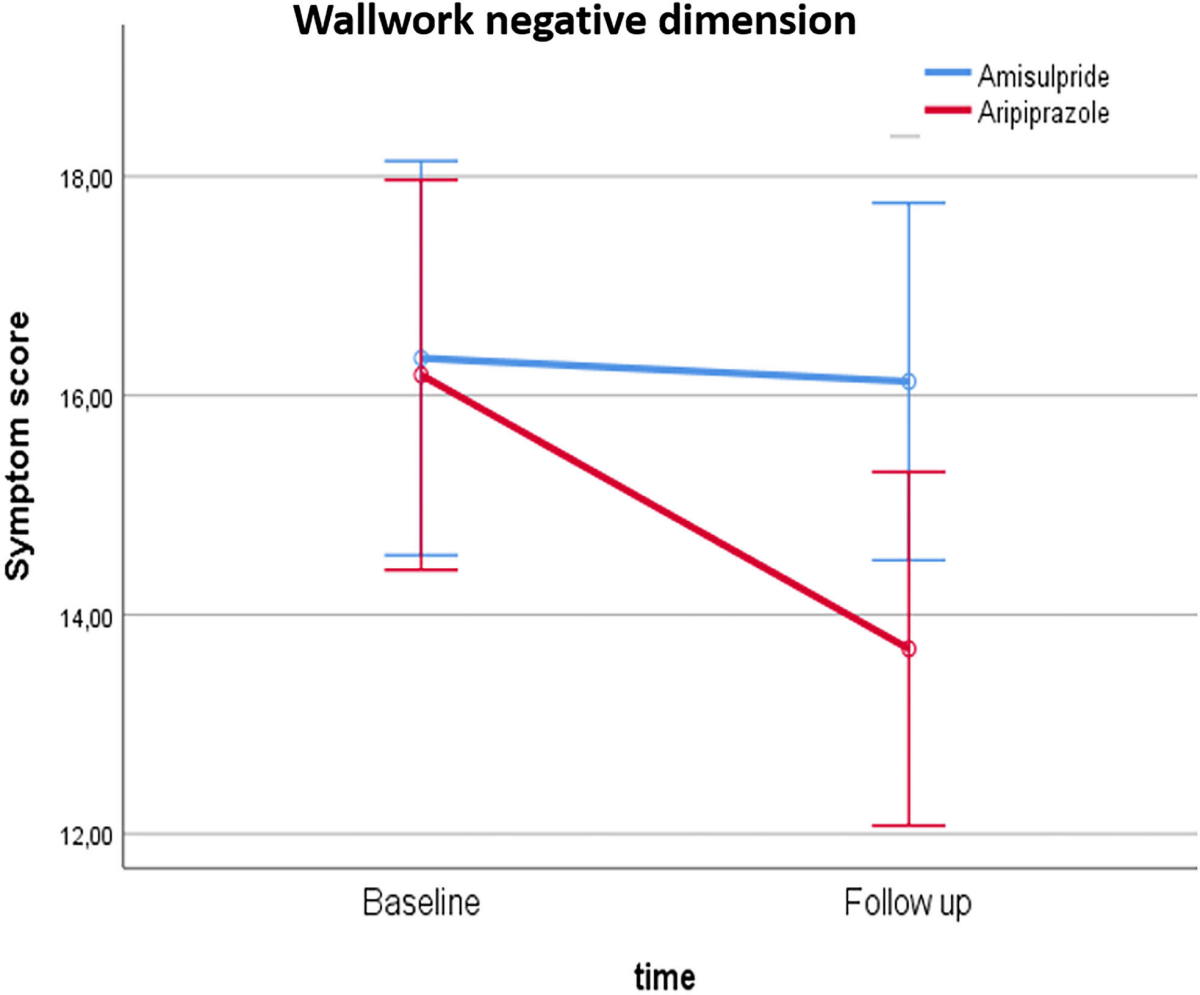
Illustration of the group*time interaction on the Wallwork negative symptom dimension score.

For the primary outcome, the Wallwork negative dimension, repeated-measure ANOVA showed a cohort^*^time interaction [*F*_(1, 92)_ =4.29, *p* = 0.041] and a significant effect of time [*F*_(1, 92)_ = 6.033, *p* = 0.016] but no effect of cohort (*p* = 0.235). *Post hoc* analyses showed a difference between cohorts after 6 weeks [*t*_(92)_ = 2.11, *p* = 0.037], which was not found at baseline (*p* = 0.93) and a paired sample *t*-test showed an effect of time in the cohort treated with aripiprazole [*t*_(47)_ =4.1, *p* < 0.001], but not in the cohort treated with amisulpride (*p* = 0.23), illustrated in Figure 2. Including sex and age as covariates made the cohort^*^time interaction slightly more significant [*F*_(1, 92)_ = 5.54, *p* = 0.021]. There was no primary effect of either sex or age, but the effect of time disappeared, and a sex–time interaction was found [*F*_(1, 92)_ =4.21, *p* = 0.043]. Men improved in Wallwork negative symptoms score in both cohorts, whereas women improved on aripiprazole but worsened on amisulpride although none of these *post hoc* results reached significance.

The additional analyses on PANSS total and PANSS positive, negative, and general subscores, showed an effect of time and no effect of cohort and no cohort^*^time interaction although a trend was found for general and negative symptoms. All the Wallwork dimensions showed an effect of time: An effect of cohort was found in excitedness and disorganization with higher levels in the amisulpride cohort (Table 2). Both cohorts improved significantly in GAF score (*p* < 0.001), but there were no effects of cohort or cohort^*^time interactions. Likewise, there were no cohort^*^time interactions on ESRS score, but an overall effect of time (*p* = 0.034) and cohort (*p* = 0.018) was found. Both cohorts increased in ESRS score during treatment, and the amisulpride cohort had a higher rating at both baseline and follow up although this was not significant in the *post hoc* analyses (all *p*s > 0.09). Regarding weight and BMI, there was a cohort^*^time interaction (*p* ≤ 0.001), an effect of time (*p* < 0.001), and for BMI also an effect of cohort (*p* = 0.048). The weight increase was driven by patients treated with amisulpride; these patients had a higher weight and BMI already at baseline although this was not significant (*p* > 0.10).

**Table 2:** Psychopathology, side effects and level of function for both cohorts at baseline and after six weeks.

**Variable**	**Amisulpride 47**		**Aripiprazole 48**		**ANOVA, p-value**		
	**Baseline**	**Six weeks**	**Baseline**	**Six weeks**	**Time**	**Cohort**	Cohort*time
**Wallwork**							
Negative	16.3	*16.1*	16.2	*13.7*	***0.016***	0.235	***0.041***
Positive	12.4	7.9	12.9	9.4	*** <0.001***	0.065	0.083
Disorganized	8.8	7.5	7.4	5.7	*** <0.001***	***0.003***	0.412
Excited	7.4	6.0	6.1	5.2	*** <0.001***	***0.018***	0.352
Depressed	10.0	7.0	9.9	7.7	*** <0.001***	0.552	0.156
**PANSS**							
Total	80.3	63.9	74.4	60.1	*** <0.001***	0.076	0.449
Positive	20.2	14.1	18.6	13.8	*** <0.001***	0.219	0.150
Negative	19.9	*19.3*	19.2	*16.5*	***0.002***	0.158	0.087
General	*40.2*	30.7	*36.6*	29.8	*** <0.001***	0.083	0.071
ESRS	3.9	5.8	2.7	3.5	***0.034***	***0.018***	0.744
GAF	41.3	54.1	46.7	54.1	*** <0.001***	0.063	0.410
Weight	77.7	80.2	71.6	71.7	*** <0.001***	0.104	***0.001***
BMI	25.3	26.1	23.8	23.9	*** <0.001***	***0.048***	*** <0.001***

*Wallwork the five dimensions from Wallwork five factor model; PANSS, Positive And Negative Syndrome Scale; ESRS, The extrapyramidal Symptom Rating Scale; GAF, Global Assessment of Function; BMI, Body Mass Index. Bold italics indicate p-values < .05*.

### Cognitive Measures

**Figure 3:**
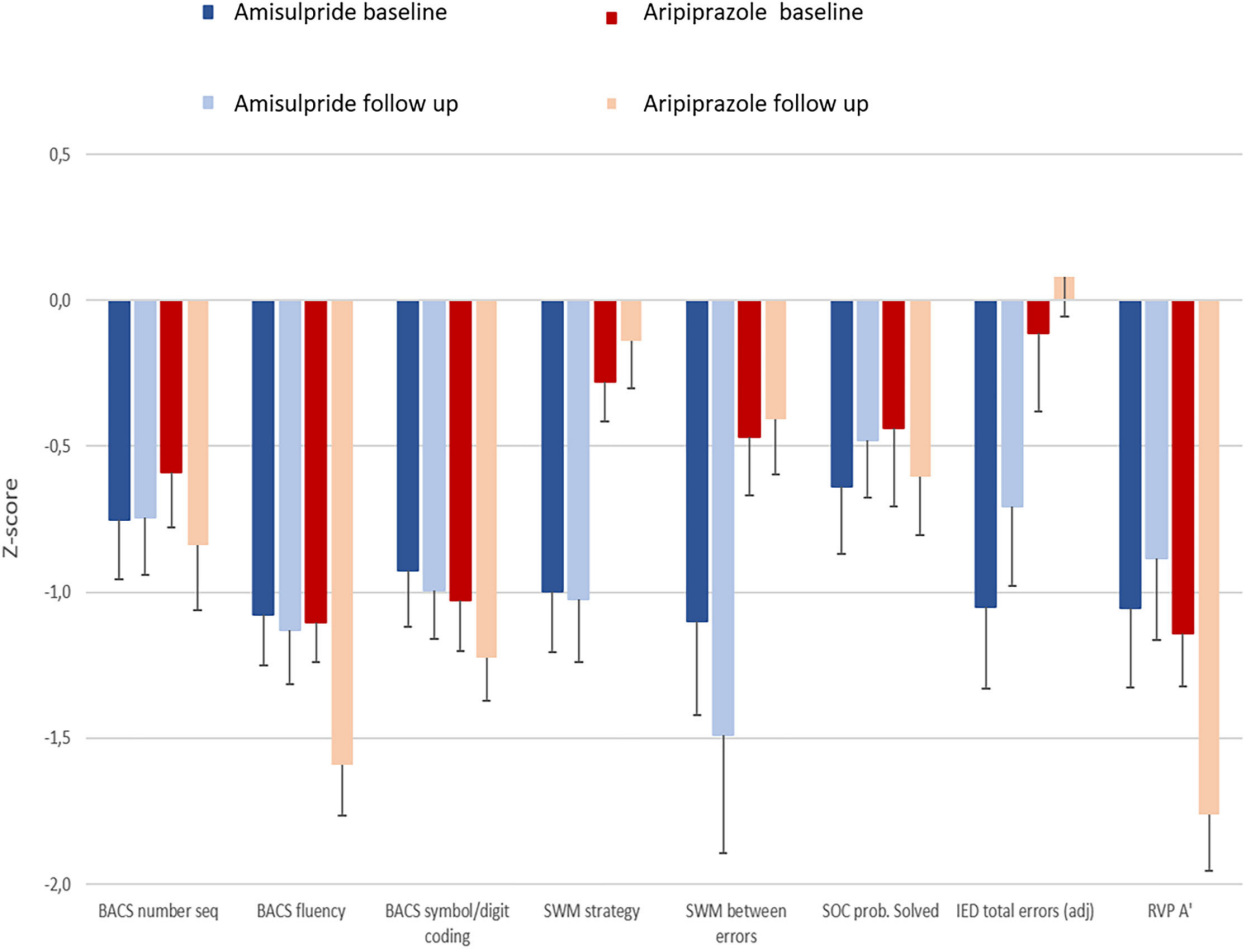
Bar graph illustrating cognitive performance for patients in both cohorts at baseline and after 6 weeks as measured by *z*-score.

For the secondary outcome, i.e., the eight selected cognitive measures, no cohort^*^time interaction survived the corrected significance threshold (*p* < 0.006). A main effect of time was found for verbal fluency (*p* = 0.002) and sustained attention (*p* < 0.001), where average *z*-scores became more negative, meaning that patients improved less after six weeks than HC. A main effect of cohort was found for spatial working memory strategy (*p* = 0.002) and at the trend level for mental flexibility (*p* = 0.008); for both measures the aripiprazole cohort had lower *z*-scores at both time points than the amisulpride cohort (Figure 3; Supplementary Table S1).

### Mixed Modeling

For the primary outcome, the Wallwork negative dimension, there was a main effect of time and no effect of cohort, but a trend-level cohort^*^time interaction (*p* = 0.067) (Table 3). For the remaining analyses, please refer to Table 4.

**Table 3:** Estimated means and *p* values from the mixed modeling analyses on the whole sample.

**Variable**	**Amisulpride** ***n*** **=** **63**		**Aripiprazole** ***n*** **=** **66**		**Mixed modeling**, ***p*****-value**		
	**Baseline**	**Six weeks**	**Baseline**	**Six weeks**	**Time**	**Cohort**	Cohort*time
**Wallwork**							
Negative	16.6	16.2	16.2	13.8	***0.007***	0.144	0.067
Positive	12.2	7.8	12.8	9.5	*** <0.001***	***0.017***	0.073
Disorganized	9.0	7.7	7.6	5.9	*** <0.001***	***0.001***	0.369
Excited	7.9	6.2	6.2	5.3	*** <0.001***	***0.001***	0.142
Depressed	10.2	7.1	10.2	7.9	*** <0.001***	0.384	0.179
**PANSS**							
Total	83.0	65.4	75.7	60.7	*** <0.001***	***0.013***	0.302
Positive	20.4	14.2	18.7	13.8	*** <0.001***	0.106	0.114
Negative	20.6	19.5	19.4	16.7	*** <0.001***	0.050	0.158
General	41.9	31.7	37.5	30.2	*** <0.001***	***0.016***	***0.046***

*Bold italics indicate p-values < .05*.

**Table 4:** Baseline psychopathology score on patients with and without follow up data and p-values for ANOVA comparing the four groups.

**Variable**	**Amisulpride**		**Aripiprazole**		
	**Stayed** ***N* = 47**	**Dropped out** ***N* = 16**	**Stayed** ***N* = 48**	**Dropped out** ***N* = 18**	**ANOVA** ***p*-value**
**Wallwork**					
Negative	16.3	18.1	16.5	16.1	0.761
Positive	12.4	11.8	12.9	12.5	0.529
Disorganized	8.8	10.0	7.4	8.7	***0.025***
Excited	7.4	9.4	6.1	6.7	***0.001***
Depressed	10.0	10.6	9.9	11.1	0.387
**PANSS**					
Total	80.3^*^	90.9^*^	74.4	79.3	***0.003***
Positive	20.2	21.1	18.6	18.9	0.130
Negative	19.9	23.4	19.2	20.1	0.172
General	40.2^*^	46.5^*^	36.6	40.3	*** <0.001***

* *Indicate group difference at baseline (p < 0.05). Bold italics indicate p-values < .05*.

Importantly, including the patients who dropped out introduced a main effect of cohort in PANSS total and general score, which was not observed in the original analyses. This was confirmed by dropout analyses showing a difference in baseline PANSS total and general score and in the disorganized and excitement dimension on the Wallwork five-factor model. Except for the Wallwork positive dimension, patients who dropped out had a higher baseline psychopathology score. This was most pronounced in the amisulpride cohort, where a *post hoc t*-test showed a significant difference for PANSS total and general score (both *p*-values < 0.02, all other *p*-values < 0.05).

Performing mixed modeling analyses on the cognitive measures resulted in results identical with the primary analyses: No cohort^*^time interaction survived the corrected significance threshold (*p* < 0.006). A main effect of time was found for verbal fluency (*p* = 0.003) and sustained attention (*p* < 0.001). A main effect of cohort was found for spatial working memory strategy (*p* = 0.002) and at the trend level for mental flexibility (*p* = 0.031, Supplementary Table S2).

Removing the 13 patients with other psychoses diagnoses resulted in less comparable groups as a group difference in baseline psychopathology was introduced (Supplementary Table S4). Further, removing 27% of the data in one of the cohorts reduced the power for detecting significant development over time. Therefore, these analyses are only presented in the Supplementary Material.

## Discussion

In the present analyses examining patients with first-episode psychoses who had not previously been treated with antipsychotic medication, we found a significant decrease in negative symptoms of 2.5 points in patients treated with aripiprazole for 6 weeks but not in patients treated with amisulpride. We found no indication of any positive effects on the cognitive performance of the two antipsychotic compounds when controlling for simple retest effect. In addition, we found a significant weight gain in the amisulpride cohort, whereas the aripiprazole cohort were weight stable during these first 6 weeks of antipsychotic treatment.

The primary aim of the presented analyses was to compare the effect on negative symptoms of two antipsychotic drugs that are both recommended for first-line treatment in patients with first-episode psychoses. Treating negative symptoms is relevant because the level of negative symptoms has a high impact on the long-term outcome (47, 48). Although amisulpride in low doses (<300 mg) is registered for treatment of negative symptoms in Denmark, we were not able to measure a treatment effect on any of the negative symptom dimensions we analyzed. This was the case although patients were treated with relatively low doses, and therefore, they did not develop extrapyramidal side-effects (EPS), which could have induced secondary negative symptoms and affected their negative symptom score. The fact that there were no significant development of EPS and no group^*^time interaction on this measure is important because most previous studies compare aripiprazole with compounds such as haloperidol and risperidone, which are prone to induce EPS (21–24, 28). Our results indicate that the superior effect of aripiprazole on negative symptoms is not only accounted for by not inducing EPS. One could argue that ESRS only measures EPS, whereas it does not specifically address other important side effects such as feeling or being sedated. We can, therefore, not rule out that different level of sedation in the two cohorts may explain some of the difference in the negative symptom score.

Importantly, patients treated with amisulpride improved just as much on positive and general symptoms as the patients treated with aripiprazole, which indicates that the difference in treatment effect is not accounted for by an effect on secondary negative symptoms, such as being socially isolated because of anxiety or psychotic symptoms. This could indicate that aripiprazole due to the dopamine receptor agonistic properties has an effect on primary negative symptoms although primary negative symptoms are difficult to disentangle from secondary negative symptoms, especially in recently diagnosed first-episode patients. There is, however, other evidence pointing toward third-generation antipsychotics that may influence primary negative symptoms. In a recent study focusing specifically on patients with primary negative symptoms, an effect of cariprazine was found on several different PANSS-derived factors (49). Future studies, including neurophysiological measures of specific neurocircuits while examining change in negative symptoms during treatment with a partial dopamine receptor agonist, may be able to establish a direct link between the influence on neurophysiology and negative symptoms.

Because the patients were all first-episode psychotic patients, we chose the Wallwork definition of the negative symptom dimension of PANSS items (39). It would have been optimal to use one of the newer negative symptom rating scales, such as the Brief Negative Symptom Scale (BNSS) (50) or the Clinical Assessment Interview for Negative Symptoms (CAINS) (51). Unfortunately, the data were collected in the period 2009–2019, when these scales were being developed, and the BNSS were not translated into Danish and validated in a Danish sample until 2019 (52). In future studies, it would be highly relevant to examine the effect of dopamine receptor agonists on negative symptoms using one of the new scales, in which also the effect on different subdomains could be explored.

We did not observe a treatment effect on any of the cognitive measures. This is interesting because the design of the present study corrected for the retest effect by calculating *z*-scores based on healthy controls examined at the same time point. Previous studies did not use this strategy. Some studies only included one group, and thus, any improvement may simply be a retest effect (25, 27). Other studies compared two groups of patients receiving different antipsychotics in which any group difference may reflect differences in retest effect rather than an actual improvement in cognitive functions (26, 28). Thus, the clinical evidence of aripiprazole having a superior effect on cognitive deficits is not convincing. Nonetheless, there is limited evidence in humans that a partial D2 receptor agonist can at least affect working memory. One study on seven patients with schizophrenia found a relation between the D2 receptor occupancy of aripiprazole in striatum and the performance on an *N*-back test (53). The occupancy in prefrontal cortex was not directly measured, but the authors assumed that the result could be extrapolated to include prefrontal cortex. Whether this is plausible can be debated, but the results are interesting and underline the importance of addressing this directly in future imaging studies.

Although metabolic issues were not a primary or secondary outcome in the present study, it is important to notice that we observed no weight gain in the cohort treated with aripiprazole, whereas this was the case for patients treated with amisulpride. Although we only collected data during the first 6 weeks of treatment, this is an important observation because metabolic side effects constitute a major clinical problem.

In our primary analyses, we did not include patients who dropped out of the studies. Including these patients by using mixed modeling changed our results on psychopathology slightly, and the different effect on negative symptoms was now only a trend. However, dropout analyses showed that psychopathology at baseline in the patients who dropped out differed between the two cohorts. Including these patients in the analyses decreased cohort comparability and may, therefore, not be the optimal approach for these data. Also, it is important to note that comparing the treatment effect between cohorts was not a main aim of the original studies, which is, of course, a major limitation. The data were collected consecutively, unblinded, and there were diagnostic differences between cohorts. Although collected consecutively, the data was collected by the same research group, which may decrease variability in rating traditions. Because the present analyses were not planned when any of the studies were carried out, raters were not biased toward one of the compounds. A randomized design would be optimal although the two cohorts were very similar regarding age, sex, level of symptoms, and functioning. Importantly, diagnostic differences are present: The amisulpride cohort only included patients with schizophrenia/schizoaffective psychoses, whereas 27% of the patients in the aripiprazole cohort had other psychoses diagnoses. One could argue that the subgroup with other psychoses diagnosis may not have the same level of negative symptoms because negative symptoms do not appear in the diagnostic criteria. This was not the case in our additional analyses, where we found that the cohorts became less comparable regarding psychopathology after excluding these patients. We do, therefore, not believe that the diagnostic difference explains the effect of aripiprazole on negative symptom. We chose to use the Wallwork negative symptom dimension because this has been suggested in literature (40). This dimension does, however, include motor retardation, which other guidelines recommend should be excluded (54). The use of Wallwork and not one of the newer scales is a limitation.

Negative symptoms and cognitive deficits remain a challenge in the treatment of psychosis, and so far, there are no medical treatment strategies showing convincing effect. Although we found no effect on cognitive performance when accounting for the retest effect, our results support the notion that partial dopamine receptor agonists may improve negative symptoms in first episode psychoses patients.

## Data Availability Statement

The original contributions presented in the study are included in the article/Supplementary Materials, further inquiries can be directed to the corresponding author/s.

## Ethics Statement

The studies involving human participants were reviewed and approved by De Videnskabsetiske Komitéer, Region Hovedstaden, København. The patients/participants provided their written informed consent to participate in this study.

## Author Contributions

BG and BE initiated and designed the studies. MN, KB, and CL participated in data collection. MN, CL, and TK performed the analyses and interpreted the results. MN drafted the manuscript. All authors have revised and approved the final manuscript.

## Funding

The study was financially supported by the Lundbeck Foundation (R25-A2701 and R155-2013-16337), and the Mental Health Service in the Capital Region of Denmark. The funding sources had no role in the design and conduct of the study; the collection, management, analysis, and interpretation of the data; the preparation, the review or approval of the manuscript; or in the decision to submit the manuscript for publication.

## Conflict of Interest

BE is part of the Advisory Board of Eli Lilly Denmark A/S, Janssen-Cilag, Lundbeck Pharma A/S, and Takeda Pharmaceutical Company Ltd. and has received lecture fees from Bristol-Myers Squibb, Boehringer Ingelheim, Otsuka Pharma Scandinavia AB, Eli Lilly Company, and Lundbeck Pharma A/S. The remaining authors declare that the research was conducted in the absence of any commercial or financial relationships that could be construed as a potential conflict of interest.

## Publisher's Note

All claims expressed in this article are solely those of the authors and do not necessarily represent those of their affiliated organizations, or those of the publisher, the editors and the reviewers. Any product that may be evaluated in this article, or claim that may be made by its manufacturer, is not guaranteed or endorsed by the publisher.
