# Supplementary material for: Differential Effects of Aripiprazole and Amisulpride on Negative and Cognitive Symptoms in Patients With First-Episode Psychoses
Source: Front Psychiatry. 2022 Mar 17;13:834333. doi: 10.3389/fpsyt.2022.834333 (PMC8969108; doi:10.3389/fpsyt.2022.834333)
Supplement: Supplementary file 1 [file Data_Sheet_1.docx]

**Table S1 Cognitive performance (z-scores) for both cohorts at baseline and after 6 weeks**

| Cognition; | N | Amisulpride | | Aripiprazole | | Statistics ANOVA, p-value | | |
| --- | --- | --- | --- | --- | --- | --- | --- | --- |
| z-scores |  | baseline | Six weeks | baseline | Six weeks | time | cohort | cohort*time |
| BACS NSq | 42/38 | -.74 | -.75 | -.66 | -.83 | .538 | .811 | .511 |
| BACS VF | 42/48 | -1.13 | -1.08 | -1.25 | -1.59 | ***.002*** | .249 | *.018* |
| BACS SC | 42/46 | -.99 | -0.92 | -1.18 | -1.22 | .148 | .473 | .638 |
| SWM strategy | 42/48 | -1.0 | -1.02 | -.28 | -.14 | .737 | ***.002*** | .549 |
| SWM errors | 42/48 | -1.1 | -1.49 | -.47 | -.41 | .164 | .059 | .222 |
| SOC | 42/46 | -.48 | -.63 | -.47 | -.61 | .974 | .843 | .443 |
| IED | 40/48 | -1.1 | -0.7 | -.11 | .08 | .093 | *.008* | .397 |
| RVP’A | 37/41 | -.95 | -.94 | -.90 | -.92 | ***<.001*** | .654 | .118 |

***Corrected significance threshold; p<0.006*** *Uncorrected significance threshold; p<0.05*

BACS: Brief Assessment of Cognition in Schizophrenia

| Cognition; | Amisulpride | | Aripiprazole | | Statistics ANOVA, p-value | | |
| --- | --- | --- | --- | --- | --- | --- | --- |
| z-scores | baseline | Six weeks | baseline | Six weeks | time | cohort | cohort*time |
| BACS NSq | -.81 | -.79 | -.79 | -.87 | .520 | .962 | .402 |
| BACS VF | -1.35 | -1.39 | -1.25 | -1.70 | ***.003*** | .568 | *.018* |
| BACS SC | -.98 | -1.03 | -1.19 | -1.30 | .201 | .211 | .714 |
| SWM strategy | -1.0 | -1.03 | -.40 | -.30 | .598 | ***.001*** | .459 |
| SWM errors | -1.02 | -1.41 | -.83 | -.82 | .197 | .285 | .177 |
| SOC | -.84 | -.59 | -.47 | -.59 | .640 | .444 | .212 |
| IED | -.91 | -0.61 | -.34 | .04 | .086 | *.031* | .982 |
| RVP’A | -.78 | -1.90 | -1.20 | -1.76 | ***<.001*** | .636 | .110 |

NSq: verbal working memory numbers sequences, VF: verbal fluency SC: processing speed symbol coding, SWM: spatial working memory, strategy and between errors SOC: spatial planning, Stockings of Cambridge, IED: mental flexibility, Intra-Extradimensional Set Shifting

RVP’A: sustained attention, A’ from Rapid Visual Information processing

**Table S2 Estimated means and p values from the mixed modeling analyses on the Cognitive performance (z-scores) for the whole baseline sample**

**‘Schizophrenia only’ analyses**

|  | PECANS 1  N=47 | PECANS 2  N=48 | PECANS 2 Schizophrenia only  N=35 |
| --- | --- | --- | --- |
| Age (SD, range), years | 24.5 (6; 18-43) | 22.9 (4; 18-42) | 24.0 (5; 18-35) |
| Sex, female/male | 20/27 | 24/24 | 18/17 |
| Dose , mg (SD, range) | 276 (173; 50-800) | 10 (4.7; 2.5-25) | 11 (5.0; 2.5-25) |
| Chlorpromazine equivalent, mg | 216 (124; 37.5-600) | 201 (94; 50-500) | 219 (100; 50-500) |
| Diagnoses |  |  |  |
| Schizophrenia | 45 | 34 | 34 |
| Persistent delusional disorder |  | 2 |  |
| Scizoaffective psychoses | 2 | 1 | 1 |
| Other non-organic psychotic disorders |  | 8 |  |
| Unspecified non-organic psychotic disorders |  | 3 |  |

After excluding the 13 patients with other psychoses diagnosis, the groups were still comparable regarding demographic variables and medication doses (Table S3), but baseline group differences were found regarding psychopathology (Table S4)

**Table S3 Demography, antipsychotic dose, and diagnoses for both cohorts with and without patients with other psychoses diagnosis.**

For the primary outcome, the Wallwork negative symptom dimension, repeated measure ANOVA showed no effect of time (p=.078), cohort (p=.281) or cohort*time interaction (p=0.154), Post hoc analyses with paired sample t-test showed an effect of time in the cohort treated with aripiprazole (t34=2.89, p=.007), but not in the cohort treated with amisulpride (p=.82). There was no cohort difference at either baseline (p=.732) or after six weeks (p=.085). Including sex and age as covariates did not significantly alter any results. Analyses on PANSS total, and PANSS positive, negative and general subscores, showed an effect of time on all items. There was an effect of cohort on PANSS total score and a cohort*time interaction was found on PANSS general score. Both cohorts improved, but the improvement was most pronounced in the amisulpride cohort. However, post hoc analyses showed higher baseline PANSS total, positive and general score in the amisulpride cohort, which was not found at follow up. At follow up the aripiprazole group had lover PANSS negative score although no difference was found at baseline. The rest of the Wallwork dimensions showed an effect of time, no cohort*time interactions, but an effect of cohort was found in level of excitedness and disorganization with higher level in the amisulpride cohort.

For the cognitive measures, no cohort*time interactions were found. There was a main effect of time for verbal fluency (p=.009) and sustained attention (p<.001). A main effect of cohort was found for spatial working memory strategy and mental flexibility (p<0.001), and at trend-level for spatial working memory between errors (p=0.018).

**Table S4 Psychopathology for both cohorts at baseline and after six weeks. For the aripiprazole cohort, data are shown for the full sample and the ‘schizophrenia only’-sample for comparability. ANOVA compare the ‘schizophrenia only’ sample from the aripiprazole cohort and the amisulpride.**

*** indicate cohort differences at baseline and follow up (aripiprazole compared to amisulpride, p<.05)**

| Variable | Aripiprazole 48 | | Aripiprazole 35 | | Amisulpride 47 | | ANOVA, p-value | | |
| --- | --- | --- | --- | --- | --- | --- | --- | --- | --- |
|  | Baseline | Six weeks | baseline | Six weeks | Baseline | Six weeks | Time | Cohort | Cohort*time |
| Wallwork |  |  |  |  |  |  |  |  |  |
| negative | 16.2 | 13.7* | 15.9 | 14.0 | 16.3 | 16.1 | .078 | .281 | .154 |
| positive | 12.9 | 9.4* | 12.9 | 9.7* | 12.4 | 7.9 | ***<.001*** | .061 | .066 |
| Disorganized | 7.4* | 5.7* | 6.9* | 5.4* | 8.8 | 7.5 | ***<.001*** | ***<.001*** | .504 |
| Excited | 6.1* | 5.2 | 5.8* | 4.9* | 7.4 | 6.0 | ***.001*** | ***.003*** | .445 |
| Depressed | 9.9 | 7.7. | 9.4 | 7.9 | 10.0 | 7.0 | ***<.001*** | .754 | ***.020*** |
| PANSS |  |  |  |  |  |  |  |  |  |
| total | 74.4 | 60.1 | 72.8* | 60.1 | 80.3 | 63.9 | ***<.001*** | ***.046*** | .209 |
| positive | 18.6 | 13.8 | 18.1* | 13.8 | 20.2 | 14.1 | ***<.001*** | .142 | .078 |
| negative | 19.2 | 16.5* | 18.8 | 16.7* | 19.9 | 19.3 | ***.019*** | .278 | .147 |
| general | 36.6* | 29.8 | 35.8* | 29.6 | 40.2’ | 30.7 | ***<.001*** | .052 | ***.043*** |
